# Oral Hygiene and Links to Oral Health of Patients Living in Lithuania and Abroad With an All-On-4 Restoration

**DOI:** 10.3290/j.ohpd.c_2656

**Published:** 2026-04-23

**Authors:** Evelina Daugėlienė, Rima Navickė, Šarūnė Barsevičienė, Monika Balčytienė

**Affiliations:** a Evelina Daugėlienė Dental Hygienist, Public Health Specialist, Higher Education Institution, Department of Oral Health, Faculty of Health Sciences Lithuania, Klaipėda County, Klaipėda. Study design and concept, acquisition of data, analysis and interpretation of the data, and drafting of the manuscript, and critical revision for important intellectual content.; b Rima Navickė Dental Hygienist, Higher Education Institution, Department of Oral Health, Faculty of Health Sciences Lithuania, Klaipėda County, Klaipėda. Study design and concept, acquisition of data, analysis and interpretation of the data, and drafting of the manuscript, and critical revision for important intellectual content.; c Šarūnė Barsevičienė Public Health Specialist, Higher Education Institution, Department of Oral Health, Faculty of Health Sciences Lithuania, Klaipėda County, Klaipėda. Study design and concept, acquisition of data, analysis and interpretation of the data, and drafting of the manuscript, and critical revision for important intellectual content.; d Monika Balčytienė Public Health Specialist, Higher Education Institution, Department of Oral Health, Faculty of Health Sciences Lithuania, Klaipėda County, Klaipėda. Data analysis and interpretation, and critical revision for important intellectual content.

**Keywords:** All-on-4 restorations, oral hygiene habits

## Abstract

**Purpose:**

Tooth loss plays an important role in human health, causing functional, aesthetic, and social damage. In adults, tooth loss is an indicator of poor oral hygiene, mainly caused by dental caries and periodontal disease, or may result from congenital conditions, injury, or trauma. There is currently a rapidly growing interest in the All-on-4 restorations. The aim of the study was to assess oral hygiene and related oral health in the Lithuanian and European-based cohorts with All-on-4 restorations.

**Methods and Materials**: An observational, cross-sectional survey study was chosen for the research. A questionnaire with 32 questions was applied. The number of research participants was 125. The inclusion criteria: patients aged 18 years and older; Lithuanians and non-Lithuanians with All-on-4 restorations; patients who voluntarily agreed to participate in the study.

**Results:**

Gingival retraction and bleeding were more prevalent in the Lithuanian cohort. A total of 58.5% of participants in the Lithuanian cohort and 60% in the European-based cohort reported having been informed about potential implant-related complications associated with poor oral hygiene. 46.2% of the Lithuanian cohort and 26.7% of the European-based cohort believed they possessed sufficient knowledge regarding the care of fixed prostheses on implants.

**Conclusions:**

More patients in the Lithuanian cohort were informed about the importance of oral hygiene, and they more often think they have sufficient knowledge about the care of fixed prostheses on implants than patients in the European-based cohort.

Tooth loss and restoration of dental function with implants have been extensively studied worldwide. The main indicator for tooth loss is poor oral hygiene. Of course, there are more reasons for tooth loss, such as injury, trauma, or congenital conditions.^17,22^ Full dentures fixed on four dental implants are a modern dentistry option, allowing full restoration of chewing function and the creation of an aesthetically pleasing smile.^20^ In some cases of completely edentulous patients, implant-supported prosthesis treatment is almost impossible without complex techniques such as nerve transposition and grafting in the posterior maxilla and mandible. A solution for such situations is the All-on-4® concept. The ‘All-on-4’ treatment concept was developed by Paulo Malo with straight and angled multi-unit abutments to provide edentulous patients with an immediately loaded full-arch restoration with only four implants.^27^ The main reasons why patients turn to implantologists are the desire to have an aesthetically pleasing smile and to restore impaired chewing function. However, perfect implant placement and fixation are not enough to ensure that patients can enjoy a lifetime of restored chewing function and an aesthetically pleasing smile. The number of biological complications associated with dental implants is increasing.^26^ Good plaque control skills are crucial factors for preventing both peri-implant mucositis and peri-implantitis.^30^ Dental implants require special care, and improper cleaning of implant-supported prostheses leads to the development of gingival inflammation around the implants. Peri-implant diseases leading to implant complications (peri-implantitis and peri-implantositis) are mainly caused by plaque accumulation on the surface of fixed prostheses and the implant/support.^30^ Many patients think that implant-retained dentures do not need to be maintained, do not decay, and do not hurt, and that individual and professional oral hygiene is therefore unnecessary.^23^ However, the reverse is true: fixed prostheses are artificial, tend to accumulate more soft and pigmented plaque, and retain more food debris underneath.^3^ Patients with inadequate oral hygiene had a higher incidence of perimucositis and peri-implantitis, and their knowledge of oral hygiene with the All-on-4 restorations and their association with biological complications was very poor.^8^ Perimucositis occurs in 19% to 65% of patients who have dental implants, and peri-implantitis occurs in 1% to 47% of patients who have dental implants,^18^ other study shows that the overall incidence of perimucositis and peri-implantitis was 45.0% and 9.7%, respectively.^31^ Inflammation and bacterial infections of the surrounding tissues around implants are the most common causes of implant loss.^12^ Good oral hygiene and regular visits to oral health professionals are essential to ensure the longevity of fixed prostheses and dental implants.^15^ Appropriate oral hygiene education and motivational tools help patients to become more focused and understand why oral hygiene is so important with the All-on-4 restorations.^28^ With the All-on-4 restorations, regular professional oral hygiene is essential.^6^

The aim of this study was to assess oral hygiene and related oral health in the Lithuanian and European-based cohorts with All-on-4 restorations. They were interviewed between 28/02/2024 and 03/04/2024.

## METHODS AND MATERIALS

An observational, cross-sectional survey study was chosen. The sample consisted of 125 people, patients from various dental clinics, and they were given a link to an online questionnaire. The inclusion criteria were as follows: patients aged 18 years and older; Lithuanians and non-Lithuanians with All-on-4 restorations; and patients who voluntarily agreed to participate in the study. And the exclusion criteria were: patients under 18 years old; patients without the All-on-4 restorations; patients who don’t agree to answer the anonymous questionnaire. The questionnaire was adapted to the different characteristics: it was written in Lithuanian and English.

The research was based on a self-created, non-validated questionnaire. It was developed by the authors of the study based on scientific sources.^1,10,12,19,20,24^ A pilot study was not conducted.

A questionnaire with 32 questions was applied. The questionnaire is divided into three parts:

The first part of the questionnaire consists of demographic questions.
The second part of the questionnaire consists of 18 questions related to patients’ knowledge of individual and professional oral hygiene and the relationship of oral hygiene to oral health with the All-on-4 restorations.
The third part of the questionnaire consists of nine questions related to the oral care habits of the respondents with the All-on-4 restorations.

According to the principle of justice, all respondents participated in the survey voluntarily.

### Statistical Analysis

Statistical analysis was performed using the statistical program SPSS 24.0. Associations between categorical variables were evaluated using the Chi-square test. When the expected frequency in any cell of the contingency table was less than 5, Fisher’s Exact test was used to ensure the validity of the *P* value. The *P* value of <0.05 was considered statistically significant.

## RESULTS

### Characteristics of the Study Population

The study involved 125 patients with All-on-4 restorations. Women accounted for 53.6% and men 46.4%. The mean age was 51.54 years (youngest 23 years, oldest 70 years). The survey sample was stratified by the place of residence, and the results were compared between the two groups: the Lithuanian cohort (68 patients) and the European-based cohort (57 patients). There were no dropouts from the study.

### Characteristics of Prostheses for Patients with All-on-4 Restorations, Their Effects on Oral Health, and Associations With Periodontal Health

In the Lithuanian cohort, patients had undergone the All-on-4 restoration procedure at varying times, ranging from within the previous 2 years to more than 10 years ago. In the European-based cohort, patients had undergone All-on-4 restorations between 1 and 10 years.

The study showed (Table 1) that a substantial proportion of the Lithuanian cohort and of the European-based cohort had a temporary fixed prosthesis placed on the day of implantation. It also showed that many patients in both cohorts wore removable prostheses after implant placement. In the Lithuanian cohort, the most common prosthesis was acrylic, followed by zirconia monoliths and metal-ceramic prostheses. More than one-third of the Lithuanian patients did not know what type of temporary denture they had been fitted with. Acrylic dentures were also the most common type of denture in the European-based cohort, and a notable proportion of patients did not know which type of temporary denture they had.

**Table 1:** Period of fitting and type of temporary and permanent dentures in the Lithuanian and European-based cohorts, N (%)

Claims		Lithuanian cohort N (%)	European-based cohort N (%)	*P*
Period of temporary denture fitting	Temporary fixed prosthesis placed on the day of implantation	30 (46.2)	23 (38.3)	0.00
	Temporary fixed prosthesis not fitted	2 (3.1)	17 (28.3)	
	Wears a temporary removable denture	29 (44.6)	18 (36)	
	Doesn’t wear any temporary dentures	4 (6.2)	6 (10)	
Type of temporary denture	Metal-ceramic denture	4 (6.2)	–	0.001
	Zirconium monolith prosthesis	6 (9.2)	19 (31.7)	
	Acrylic prosthesis	31 (47.7)	31 (51.7)	
	Did not know	24 (36.9)	10 (16.7)	
Time limit for fitting a permanent denture	Not yet fitted	5 (7.7)	14 (23.3)	0.03
	After 3–6 months	33 (50.8)	27 (45)	
	After 6–12 months	23 (35.4)	12 (20)	
	After 12–24 months	4 (6.2)	7 (11.7)	
Type of permanent denture	Metal-ceramic denture	18 (27.7)	13 (21.7)	0.003
	Zirconium monolith denture	32 (49.2)	20 (33.3)	
	Acrylic prosthesis	10 (15.4)	6 (10)	
	Did not know	5 (7.7)	21 (35)	


In both the Lithuanian and European-based cohorts, permanent prostheses were most commonly fitted after 3–6 months or 6–12 months, a smaller proportion in both cohorts received them after 12–24 months, and only in the European-based cohort did some patients report not yet having received a permanent prosthesis. The Lithuanian patients were mainly fitted with zirconium monolith and metal-ceramic prostheses. Some patients had an acrylic prosthesis, and a small group did not know which type of prosthesis they had. For the European-based cohort, the most common prostheses were zirconium monolith and metal-ceramic. A smaller proportion had an acrylic prosthesis, and many patients did not know which type of permanent prosthesis they had.

Significant differences were found between the Lithuanian and European-based cohorts when analysing the periods of temporary and permanent prosthesis fitting and the type of prosthesis itself (Table 1).

In both the Lithuanian and European-based cohorts, the main reasons for tooth loss were periodontal disease, poor oral hygiene, and fear of dentists, with additional cases attributed to trauma and a small proportion related to congenital pathologies. The reasons for tooth loss did not differ significantly between the Lithuanian and European-based cohorts (Table 2).

**Table 2:** Causes of tooth loss in the Lithuanian and European-based cohorts, N (%)

Reasons	Lithuanian cohort N (%)	European-based cohort N (%)	*P*
Teeth were left untreated due to the fear of dentists	21 (39.6)	20 (40)	0.969
Poor oral hygiene	25 (47.2)	22 (44)	0.844
Periodontal diseases	33 (62.3)	29 (58)	0.691
Injuries	11 (20.8)	6 (12)	0.292
Congenital pathologies	5 (9.4)	1 (2)	0.206


In both the Lithuanian and European-based cohorts, most patients reported difficulty chewing and oral pain before the All-on-4 restorations, many experienced bleeding, some had difficulties with personal oral hygiene, and only a small number reported feeling ashamed of their dental condition. When comparing these difficulties between the Lithuanian and European-based cohorts, bleeding was a more frequent complaint among the European-based cohort (Table 3).

**Table 3:** Difficulties experienced in the Lithuanian and European-based cohorts before All-on-4 restorations, N (%)

Difficulties	Lithuanian cohort N (%)	European-based cohort N (%)	*P*
Difficulty chewing	45 (84.9)	41 (82)	0.793
Pain in the oral cavity	38 (71.7)	43 (86)	0.095
Bleeding	28 (52.8)	38 (76)*	0.023
Difficulty with oral hygiene	17 (32.1)	18 (36)	0.684
A sense of shame	5 (9.4)	2 (4)	0.438
* *P* < 0.05, compared to the Lithuanian cohort.			

The Lithuanian cohort most often chose All-on-4 restorations because everything is done in one procedure, because of the long-lasting results and because of the ability to enjoy a smile in one day. Similar reasons were reported in the European-based cohort, namely that the procedure is completed in one stage, the possibility of enjoying a smile in one day and the expectation of long-lasting results.

**Fig 1:**
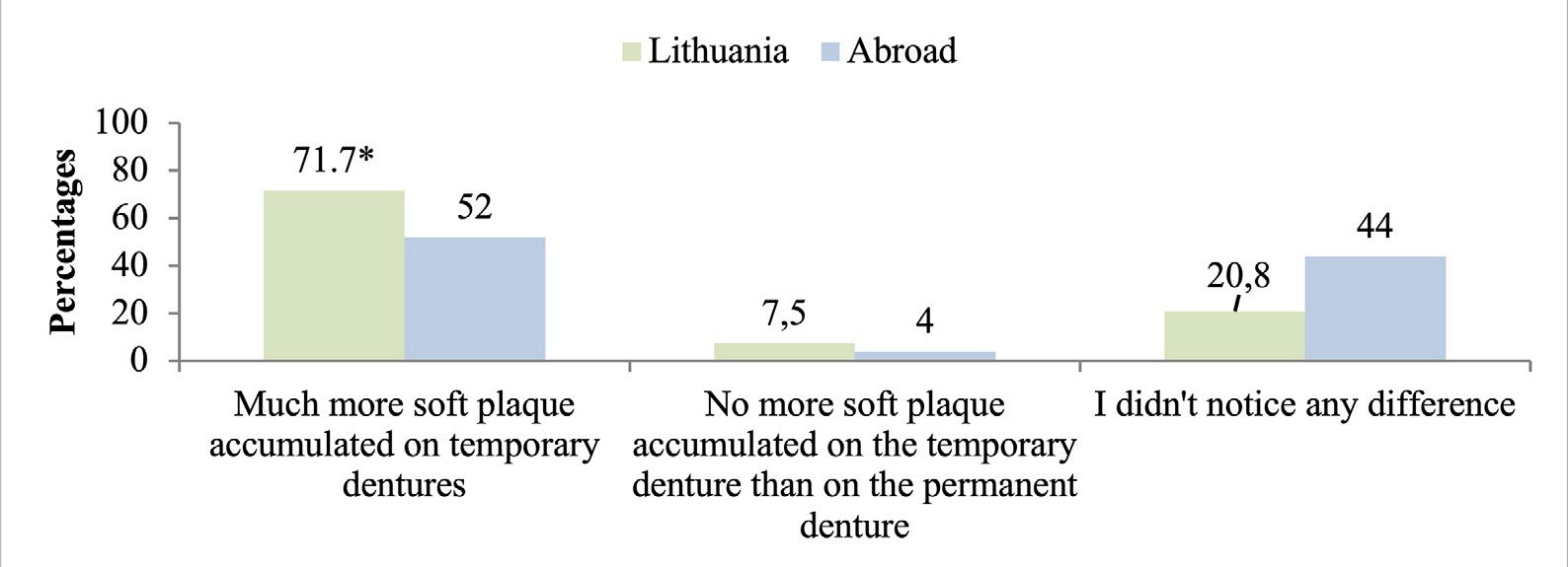
Differences in plaque accumulation between temporary and permanent dentures. * P < 0.05, compared to those who didn’t notice any difference.

The results showed (Fig 1) that the Lithuanian cohort was significantly more likely to notice a significant accumulation of soft plaque on the provisional denture than the European-based cohort, who more often reported that they did not notice a significant difference.

The results also showed (Table 4) that patients were significantly more likely to report differences in plaque accumulation between temporary and permanent dentures. In particular, patients with metal-ceramic or monolithic zirconia permanent dentures most often reported greater plaque accumulation on their temporary (predominantly acrylic) dentures than on their permanent ones, whereas patients with permanent acrylic dentures reported fewer differences.

**Table 4:** Differences in plaque accumulation between temporary and permanent dentures according to the type of permanent denture, N (%)

Claims		Type of permanent denture			*P*
		Metal N (%)	Zirconium monolith N (%)	Acrylic N (%)	
Have you noticed that more plaque has accumulated on the temporary denture than on the permanent fixed denture?	Much more soft plaque accumulated on the temporary dentures	16 (51.6)	35 (67.3)	4 (25)	0.000
	No more soft plaque accumulated on the temporary denture than on the permanent denture	2 (6.5)	5 (9.6)	4 (25)	
	I didn’t notice any difference	13 (41.9)	12 (23.1)	8 (50)	


A higher prevalence of gingival retraction was observed in the Lithuanian cohort compared with the European-based cohort. In addition, gingival bleeding was more frequent in the Lithuanian cohort than in the European-based cohort (Table 5).

**Table 5:** The prevalence of biological complications in the Lithuanian and European-based cohorts, N (%)

Complications	N	Lithuanian cohort N (%)	European-based cohort N (%)	*P*
Soft tissue exudation	9	3 (33.3)	6 (66.7)	0.245
Gingival retraction	8	7 (87.5)	1 (12.5)*	0.038
Abscess near the implant	11	5 (45.5)	6 (54.5)	0.649
Bone loss around the implant	17	11 (61.1)	7 (38.9)	0.403
Bleeding	16	12 (75.0)	4 (25.0)*	0.049
Abscess around fixed prostheses on the implants	2	0	2 (100)	0.138
*P < 0.05, compared to the Lithuanian cohort.				

Analysis of the prevalence of biological complications according to the type of permanent prosthesis revealed notable differences (Table 6). Patients with metal-ceramic prostheses showed a significantly higher frequency of bleeding around implant-supported fixed prostheses than patients with acrylic and zirconia monolith prostheses.

**Table 6:** The prevalence of biological complications among patients, depending on the type of permanent prosthesis, N (%)

Complications	N	Type of permanent prosthesis				*P*
		Metal ceramics N (%)	Zirconium monolith N (%)	Acrylic N (%)	Doesn’t know N (%)	
Soft tissue exudation	8	0	4 (50)	0	4 (50)	0.066
Gingival retraction	8	4 (50)	2 (25)	2 (25)	0	0.139
Abscess near the implant	11	4 (36.4)	4 (36.4)	0	3 (27.3)	0.455
Bone loss around the implant	18	8 (44.4)	3 (16.7)	2 (11.1)	5 (27.8)	0.065
Bleeding	16	9 (56.3)	4 (25) *	2 (12.5) *	1 (6.3) *	0.016
Abscess around fixed prostheses on the implants	8	0	2	0	0	0.430
* *P* < 0.05, compared to metal ceramics.						

### Awareness of the Oral Hygiene of All-on-4 Restorations Among the Lithuanian and European-based Cohorts

The patients living in Lithuania most often received information on the proper care of fixed dentures from the prosthetic specialists (60.4%), a professional oral hygienist (50.9%), and dental assistants (32.1%). In addition, 20.8% found the information themselves and 15.1% got it from friends or acquaintances. The European-based cohort patients most often received information from professional oral hygienists (62%), prosthetic specialists (50%), and dental assistants (38%). In addition, 28% found the information themselves, and 20% from friends and acquaintances. Based on the analysis of the results, it can be concluded that both cohorts obtain information from the same sources.

Of the Lithuanian cohort, 61.5% had been instructed and shown how to care for fixed prostheses on the implants. Also, one-fifth (23.1%) of patients were instructed but not shown, and 9.4% were not taught. The European-based cohort was slightly less likely to have been instructed and shown about the care of fixed prostheses (41.7%). 30% of them were only instructed, and 28.3% were neither shown nor instructed (compared to 15.4% in Lithuania).

It was found that the majority of the patients in both cohorts had been informed about the possible complications of implants associated with poor oral hygiene. However, a significantly higher proportion of the Lithuanian cohort than the European-based cohort reported being informed about the recommended frequency of professional oral hygiene procedures. The results showed that a larger proportion of the Lithuanian cohort than the European-based cohort had sufficient knowledge about the care of fixed prosthesis on implants, and this difference between the groups was statistically significant. However, a considerable proportion of participants in both groups reported uncertainty regarding whether their knowledge about prosthesis care was sufficient. A great proportion of patients in both cohorts considered biological complications to be related to oral hygiene. (Table 7)

**Table 7:** Awareness of the oral hygiene of All-on-4 restorations among the Lithuanian and European-based cohorts, N (%)

Claims		Lithuanian cohort N (%)	European-based cohort N (%)	*P*
Been instructed/shown how to care for implant-retained complete dentures	Yes, instructed and shown	40 (61.5)	25 (41.7)	0.069
	Instructed but not shown	15 (23.1)	18 (30)	
	No	10 (15.4)	17 (28.3)	
Been informed about the possible complications of implants due to poor oral hygiene	Yes	38 (58.5)	36 (60)	0.861
	No	27 (41.5)	24 (40)	
Been informed how often they should have professional oral hygiene	Yes	47 (72.3)	30 (50) *	0.010
	No	18 (27.7)	30 (50)	
They think they have enough knowledge about the care of fixed prostheses on implants	Yes	30 (46.2)	16 (26.7) *	0.030
	No	5 (7.7)	12 (20) *	
	They don’t know	30 (46.2)	32 (53.3)	
They think that biological complications can be linked to oral hygiene	Yes	32 (49.2)	25 (41.7)	0.136
	No	9 (13.8)	17 (28.3)	
	They don’t know	24 (36.9)	18 (30)	
* *P* < 0.05, compared to the Lithuanian cohort.				

Of the Lithuanian cohort (Table 8), 6.2% think that the oral cavity should be cleaned after every meal with the All-on-4 restorations (26.7% of the European-based cohort), and 55.4% think that it should be cleaned twice a day (36.7% of the European-based cohort), while 18.5% think that brushing once a day is sufficient (16.7% of the European-based cohort) (*P* < 0.05).

**Table 8:** Awareness of the oral hygiene of All-on-4 restorations among the Lithuanian and European-based cohorts, N (%)

Claims		Lithuanian cohort N (%)	European-based cohort N (%)	*P*
How often do they think they should brush their mouth with the All-on-4 restorations	After every meal	4 (6.2)	16 (26.7) *	0.014
	Two times a day (morning and evening)	36 (55.4)	22 (36.7)	
	Twice a day and after every meal	13 (20)	12 (20)	
	Once a day	12 (18.5)	10 (16.7)	
How long do they think it takes on average to clean an All-on-4 restorations	About 1 minute	4 (6.2)	16 (26.7) *	0.008
	2-3 minutes	31 (47.7)	23 (38.3)	
	More than 3 minutes	24 (36.9)	13 (21.7) *	
	They don’t know	6 (9.2)	8 (13.3)	
Do they think that the All-on-4 restorations require special care and individual hygiene compared to natural teeth	Requires much more care than natural teeth	18 (27.7)	15 (25)	0.431
	Same care as natural teeth	34 (52.3)	27 (45)	
	Very low maintenance	13 (20)	18 (30)	
* *P* < 0.05, compared to the Lithuanian cohort.				

Of the Lithuanian cohort, 47.7%, and of the European-based cohort, 38.3%, think that 2–3 min is enough to clean the All-on-4 restorations on average (*P* < 0.05). 52.3% of the Lithuanian cohort and 45% of the European-based cohort think that the All-on-4 restorations require the same care as natural teeth (*P* > 0.05).

### Oral Hygiene Habits of All-on-4 Restorations in the Lithuanian and European-based Cohorts

Oral hygiene practices were generally similar among the Lithuanian and European-based cohorts. Brushing teeth with toothpaste twice daily was reported by 84.9% of Lithuanian and 72% of the European-based respondents. Daily mouthwash use was reported by 50.9% and 64%, respectively, while brushing dental implants once daily was reported by 56.6% and 58%. A single toothbrush used once per day was reported by 45.3% in the Lithuanian patients, and 42% in the European-based patients, and daily use of a water irrigator was reported by 60.4% and 62%, respectively. Tongue brushing once daily was slightly more common among the Lithuanian cohort (41.5%) than the European-based cohort (30%), although this difference was not statistically significant (*P* > 0.05). Daily dental floss use was reported by 37.7% of the Lithuanian and 44% of the European-based cohorts.

Regarding denture hygiene, participants in the Lithuanian cohort reported spending 1–2 min cleaning fixed dentures in 26.4% of cases, 3–4 min in 47.2%, and 5–6 min in 26.4%. Among the cohort living in other European countries, 40% reported spending 1–2 min, 42% spent 3–4 min, 14% spent 5–6 min, and 4% spent more than 6 min cleaning their dentures.

It was found that patients who were informed about possible complications were significantly more likely to clean their dentures for 5–6 min (32.1%) than those who were not informed (8%) (*P* < 0.05).

The Lithuanian cohort was slightly more likely to visit an oral hygienist every 6 months (54.7%) compared to the European-based cohort (34%), while a higher proportion of the latter reported never receiving professional oral hygiene (22%). Among participants who had been informed about the importance of professional oral hygiene, only 5.1 % did not utilise these services, whereas 44% of those who had not received such information did not use professional oral hygiene services (*P* < 0.001). Furthermore, patients who were not informed about possible implant complications were significantly more likely to skip a professional oral hygiene procedure (26%) compared to those who had been informed (3.8%) (*P* < 0.001).

The study further demonstrated that patients who underwent professional oral hygiene annually were significantly more likely to experience biological complications than those who received professional oral hygiene every 6 months or every 3 months. Additionally, biological complications were more frequent among patients who reported cleaning their dentures only once daily compared to those who cleaned more frequently; however, this difference was not statistically significant (Table 9).

**Table 9:** Incidence of biological complications in patients with fixed dentures according to the oral hygiene habits, N (%)

Oral hygiene habits		Biological complications		*P*
		Not N (%)	Yes N (%)	
How often do you visit an oral hygienist for professional oral hygiene?	Every 3 months and more frequently	7 (18.4)	7 (8)	0.004
	Every 6 months	13 (34.2)	44 (50.6)	
	Every 12 months	16 (42.1)	17 (19.5)	
	Never	2 (5.3)	19 (21.8)	
How often do you clean a fixed denture?	1 time per day	16 (42.1)	22 (25.3)	0.268
	1 or 2 times a day	13 (34.2)	35 (40.2)	
	2 times a day	6 (15.8)	17 (19.5)	
	2 or more times a day	3 (7.9)	13 (14.9)	


**Fig 2:**
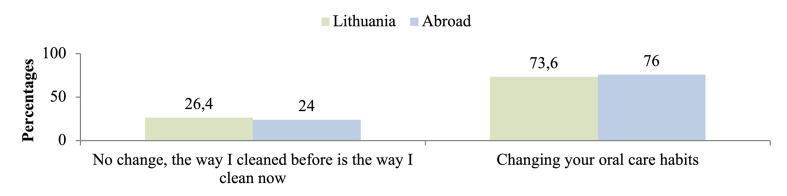
Changes in the oral hygiene habits of patients living in Lithuania and abroad after the All-on-4 restorations.

In both the Lithuanian and European-based cohorts, most participants reported modifying their oral hygiene habits after the All-on-4 restorations, while a smaller proportion in each cohort reported no change (Fig 2).

**Fig 3:**
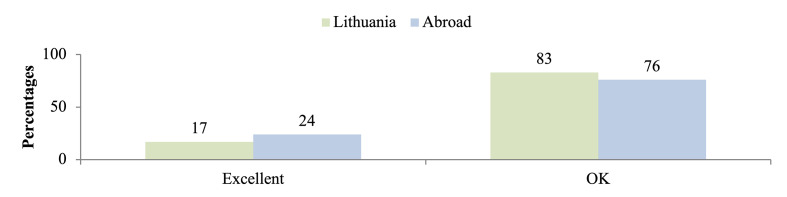
Percentage of patients living in Lithuania and abroad who rate their oral hygiene after the All-on-4 restorations.

Patients were asked to evaluate their oral hygiene after the All-on-4 restorations (Fig 3). Only a small number of patients in both cohorts rated their oral hygiene as excellent.

## DISCUSSION

The present study provides novel cross-cultural insights into patients’ perceptions, knowledge, and oral hygiene practices following All-on-4 restorations by comparing a Lithuanian cohort with a European-based cohort. One of the most important findings is that, despite comparable motivations for choosing All-on-4 treatment in both cohorts, notable differences were observed in plaque-related experiences, education pathways, and professional follow-up patterns. This highlights that patient behaviour and perceived outcomes after implant-supported full-arch restoration may be influenced not only by clinical factors but also by differences in healthcare delivery models and professional roles across countries.

Consistent with previous reports, patients in both cohorts primarily selected All-on-4 restorations because of the possibility of immediate functional and aesthetic rehabilitation and the short overall treatment time. These findings support the concept described by Fernandez-Ruiz et al, who emphasised the rapid improvement in mastication, comfort, aesthetics, and social well-being associated with this treatment approach.^14^ The high importance attributed to immediate results further underlines that expectations of rapid improvement may shape patient satisfaction and treatment decisions.

An important new observation of this study is the significantly higher frequency of self-observed soft plaque accumulation on provisional prostheses in the Lithuanian cohort compared with the European-based cohort. This finding is clinically relevant, as temporary full-arch prostheses are commonly fabricated from acrylic materials, which are known to be more susceptible to biofilm retention than definitive zirconia or metal-ceramic restorations. This observation is in agreement with previous evidence showing significantly higher plaque indices in patients wearing temporary fixed All-on-4 prostheses compared with those rehabilitated with definitive prostheses.^11^ The results underline the critical need for intensified hygiene instruction during the provisional phase of treatment.

The occurrence of biological complications reported by participants in both cohorts is consistent with earlier studies demonstrating that biological complications remain a frequent problem in edentulous patients treated with full-arch implant-supported prostheses.^9^ The presence of gingival bleeding, soft-tissue inflammation, and peri-implant problems reinforces the well-established role of bacterial accumulation around implant-supported prostheses as a key aetiological factor.^16^ The findings of the present study are in line with previous reports indicating that approximately one-third of patients experience biological complications, most commonly gingival bleeding and peri-implant bone loss.^29^

Another important aspect revealed by this study is the difference in professional sources of oral hygiene information between cohorts. The Lithuanian cohort most frequently received guidance from prosthetic specialists, whereas the European-based cohort more often received information from professional oral hygienists. Nevertheless, a substantial proportion of patients in both cohorts reported searching for information independently, which is comparable to previous studies.^5^ This highlights the need for coordinated and consistent communication between all members of the dental team, as inconsistent or fragmented advice may compromise patient understanding and adherence to recommended hygiene procedures.

Although a considerable proportion of patients reported being taught and shown how to care for fixed prostheses on implants, a notable gap between instruction and effective implementation is suggested by earlier studies, which demonstrated low adherence to recommended cleaning protocols despite high levels of reported instruction.^4,5^ The present findings support the growing evidence that providing information alone is insufficient. Patient motivation, practical training, and reinforcement through follow-up appointments are essential components of long-term implant maintenance.

Professional maintenance attendance remains suboptimal in both cohorts. While the Lithuanian cohort was more likely to attend professional oral hygiene visits every 6 months than the European-based cohort, a substantial proportion of patients – particularly within the European-based cohort – reported never receiving professional oral hygiene. This is of concern, as professional plaque control combined with patient-performed hygiene is considered the standard of care for the prevention and management of peri-implant mucositis and for reducing the risk of implant failure.^2,7^ The findings mirror previous studies reporting limited adherence to recommended professional maintenance schedules.^13,21,25^

The low proportion of participants in both cohorts who rated their oral hygiene as excellent further suggests limited confidence in personal oral care following All-on-4 restorations. Together with the reported biological complications, this indicates that current educational and maintenance strategies may be insufficient to ensure optimal long-term outcomes.

A major strength of the present study is the comparative evaluation of Lithuanian and European-based cohorts, as research investigating oral hygiene practices and perceived oral health outcomes in patients with All-on-4 restorations remains scarce, particularly in Lithuania. Cross-country comparisons in this field are also limited. By addressing this gap, the study provides new insights into how differences in professional support structures and patient education pathways may influence patient-reported outcomes.

Based on the present findings, the following recommendations for clinical practice are proposed:

Implement structured pre- and post-operative hygiene education programmes;
Use visual aids, videos, and demonstration models to explain biofilm accumulation and its associated risks, including peri-implantitis;
Encourage patients to attend professional oral hygiene visits at least twice per year, or more frequently for individuals at higher risk.

### Limitations

One of the primary limitations of this study is the small sample size. This study did not assess plaque accumulation or compliance with hygiene instructions over time, which limits the strength of causal interpretations. Furthermore, the use of a non-validated and self-created questionnaire, self-report bias, recall bias, lack of clinical examination, and potential sampling bias may affect the generalizability of results. The lack of pilot testing may impact reliability, too. The cultural and healthcare system differences between countries may also influence patient behaviour and reporting accuracy, which should be considered when interpreting the findings.

## CONCLUSIONS

Gingival retraction and bleeding were more common in the Lithuanian cohort than in the European-based cohort. Metal-ceramic prostheses cause more bleeding than acrylic or zirconia. The Lithuanian cohort mainly received oral hygiene guidance from prosthetic specialists, while the European-based cohort was advised by hygiene specialists. More Lithuanian cohort members were informed about the importance of oral hygiene than the Europe-based cohort. Informed patients were more likely to clean thoroughly. Most of the patients in both cohorts changed their oral hygiene habits after All-on-4 restorations. The increased bleeding with metal-ceramic prostheses highlights the importance of material choice.

Standardised hygiene education and follow-up protocols would: provide a uniform baseline of knowledge for patients regardless of location; help reduce disparities by ensuring that even underserved populations receive accurate and actionable oral hygiene instructions; ensure that evidence-based recommendations are consistently delivered, and dentists and hygienists follow the same scientifically validated guidelines. Overall, standardised hygiene education is vital irrespective of the treatment setting.

### Acknowledgements

#### Ethics approval and consent to participate

Ethics approval for the study was obtained from the Bioethics Committee of the Department of Oral Health (No. SSV6-27). Completion and submission of the survey were considered consent for participation in the study.

#### Data availability

The data sets generated and/or analysed during the current study are available from the corresponding author on reasonable request. This research received no external funding.

#### Conflict of interest

The authors declare no conflict of interest.
